# Immunomodulatory Effects of *Saccharomyces cerevisiae* Fermentation Product Supplementation on Immune Gene Expression and Lymphocyte Distribution in Immune Organs in Broilers

**DOI:** 10.3389/fvets.2017.00037

**Published:** 2017-03-13

**Authors:** Wen K. Chou, Jungwoo Park, John B. Carey, Don R. McIntyre, Luc R. Berghman

**Affiliations:** ^1^Department of Poultry Science, Texas A&M University, College Station, TX, USA; ^2^Diamond V Mills, Cedar Rapids, IA, USA; ^3^Department of Veterinary Pathobiology, Texas A&M University, College Station, TX, USA

**Keywords:** *Saccharomyces cerevisiae* fermentation product, immunomodulation, qPCR, spleen, thymus

## Abstract

A study was conducted to evaluate the molecular and cellular immunomodulatory effects of a *Saccharomyces cerevisiae* fermentation product (Original XPC, Diamond V) in broilers. Our lab has previously demonstrated that broilers fed XPC generate faster and stronger antigen-specific humoral immune responses to Newcastle disease virus (NDV) vaccination. This study aims at investigating the mechanism behind this increased immunocompetence. One-day-old broilers were randomly assigned to one of two treatments: 1.25 kg/ton *S. cerevisiae* fermentation product (XPC treatment group) or control diet. Birds were vaccinated against NDV on day 1 (B1 strain) and day 21 (LaSota strain) post-hatch. Innate and adaptive immune-related gene expression profiles in central (thymus and bursa of Fabricius) and peripheral (spleen) immune organs were investigated at 14 and 28 days of age by qPCR array. Fold changes larger than 1.2 (*P* < 0.05) between treated and control were considered significant. Lymphocyte subpopulations in central and peripheral immune organs and blood leukocytes were analyzed by flow cytometry at 14, 21, 28, and 42 days of age. In the spleen, Th1 immune responses and antiviral genes, such as IFN-γ, and its downstream genes signal transducer and activator of transcription (STAT4) and NFκB, were significantly upregulated in the treated group by 14 days of age. In the thymus, genes belonging to different functional groups were influenced at different time points. Cytokine genes associated with lymphocyte maturation, differentiation, and proliferation, such as IL-1R, IL-2, and IL-15 were significantly upregulated in the treated group by 28 days of age. Genes preferentially expressed in the medulla of the thymus and mature thymocytes, such as Myxovirus resistance gene 1, interferon regulatory factor-1, interferon regulatory factor-7, and STAT1, were upregulated in the birds supplemented with XPC. Birds supplemented with XPC had significantly higher percentages of CD3^+^, CD4^+^, and CD8^+^ T-cells in the thymus at day 28 of age, indicating production of more mature T-cells, which was consistent with gene expression results. Results suggest that XPC supplementation primes broilers to become more immunocompetent, without compromising growth performance.

## Introduction

Over the past five decades, production efficiency including rapid growth, superior feed conversion, and high processing yield in the poultry industry have increased dramatically due to genetic selection (1, 2). Unfortunately, genetic selection for performance traits may result in negative impacts on immunity (1, 3). In the modern poultry industry, intensive production and confined housing conditions increase the risks of exposure to pathogens and stress factors (4). Therefore, several tools, including vaccination, biosecurity, antibiotic growth promoters, and nutritional immunomodulators, have been developed in order to maintain the balance between growth performance and the animal’s health and welfare. Recently, concerns over development of antimicrobial resistance from animals to humans led to changes in legislation regarding the use of antibiotics in poultry diets. Removal of antibiotic growth promoters has become desirable in light of evolving consumer demand. Thus, efforts have been focused on development of natural immunomodulators.

The characteristics of natural immunomodulators have been discussed in detail (4, 5). Recently, the immunological effects of *Saccharomyces cerevisiae* fermentation product (Original XPC™, Diamond V; referred as XPC in the following context) have been reported in several studies. Gao et al. (6) reported that supplementation with XPC not only resulted in better average daily weight gain and feed conversion rates through the improvement of intestinal morphology but also stimulated both innate and humoral immunity. They demonstrated serum lysozyme and antigen-specific antibody titers significantly increased when broiler diets were supplemented with XPC. In the follow-up study, Gao et al. (7) further reported broilers whose diets were supplemented with XPC were able to recover body weight lost due to *Eimeria tenella* infection. XPC supplementation also increased T-cell repertoires in the spleen and peripheral blood, indicating an upregulation of cellular immunity in the XPC supplemented broilers. Similarly, Lensing et al. (8) report supplementation with XPC decreased the severity of lesions after *Eimeria maxima* challenge. Despite the lack of evidence indicating a direct anti-coccidian effect of dietary XPC, it has been suggested that XPC can help balance the immune system of broilers in an indirect manner. Supplementation with XPC also prevented absorption of aflatoxin in the intestinal tract of chickens (9). Taken together, these studies suggest that XPC supplementation is able to balance and modulate the immune system without compromising growth performance in broilers.

Our previous study also demonstrated that dietary supplementation with XPC in broilers not only significantly improved feed conversion rate but also accelerated the establishment of a Newcastle disease virus (NDV)-specific humoral immune response when compared with non-supplemented broilers (10). While the beneficial effects of XPC supplementation on broiler immunity have been convincingly documented, the impact of supplementation on the immune cell repertoire and gene expression patterns in immune organs of broilers, especially after challenge, has yet to be addressed.

In the current manuscript, we further discuss the effect of dietary XPC on cell-mediated immunity (CMI) by measuring the lymphocyte repertoire in immune organs (bursa, thymus, and spleen) and peripheral blood on 14, 21, 28, and 42 days of age using four-color flow cytometry. Also, gene expression profiling in immune organs (bursa, thymus, and spleen) was performed at 14 and 28 days of age using the innate and adaptive immune response RT^2^ *Profiler*™ PCR array (SABiosciences, Frederick, MD, USA). This array profiles 84 immune-related genes functionally grouped into innate, adaptive, humoral immunity, inflammatory response, and defense response against bacteria and viruses, which provides a comprehensive overview of the effects of XPC supplementation on an extensive array of the birds’ immune parameters upon challenge with a live-attenuated NDV vaccine.

## Materials and Methods

### Experimental Design

One hundred fifty broilers (Ross 708, Aviagen, Huntsville, AL, USA) were randomly assigned into control and treated groups at 1 day of age. The dietary treatments consisted of 0 (Control) and 1.25 kg/metric ton of *S. cerevisiae* fermentation product (XPC; Original XPC™, Diamond V, Cedar Rapids, IA, USA). Starter, grower, and finisher complete diets (Table 1) were manufactured at the Texas A&M University Poultry Research, Teaching and Extension Center. All the birds were vaccinated with 30 μL of live-attenuated NDV B1 strain vaccine (Merial, Duluth, GA, USA) on day 1 of age and boosted with live-attenuated NDV LaSota strain vaccine (Merial, Duluth, GA, USA) on 21 days of age *via* eye drop. In order to assess the effects of XPC on broiler CMI, immune organs and peripheral blood mononuclear cells (PBMC) were collected every 7 days (except for 35 days of age) from as early as 14 to 42 days of age (the end point of the experiment). In addition, gene expression profiling of immune organs was performed at 14 and 28 days of age. All research on animals reported in this manuscript was approved by the Institutional Animal Care and Use Committee of Texas A&M University.

**Table 1:** **Experimental diets and nutrient composition**.

	Starter (0–14 days)	Grower (15–28 days)	Finisher (29–42 days)
**Ingredients, %**			
Corn, yellow grain	58.46	64.73	68.53
Soybean meal, dehulled solvent	34.48	29.33	24.64
Bio Phos 16/21 P	1.56	1.59	1.29
Limestone	1.49	1.46	1.60
Salt, plain	0.51	0.49	0.46
Mineral mix^a^	0.05	0.05	0.05
dl-Methionine	0.28	0.08	0.16
Lysine	0.17	0.02	0.12
Vitamin premix^b^	0.25	0.25	0.25
Fat, A/V blend	2.75	2.00	2.90
Original XPC^c^	0.125	0.125	0.125
**Nutrient composition**			
Crude protein, %	22.02	19.80	17.99
ME, kcal/kg	3,048	3,055	3,151
Crude fat, %	5.30	4.74	5.73
Lysine, %	1.30	1.05	1.00
Methionine (M), %	0.61	0.39	0.44
M + Cysteine, %	0.97	0.72	0.75
Tryptophan, %	0.26	0.23	0.20
Threonine, %	0.82	0.74	0.67
Arginine, %	1.45	1.29	1.14
Valine, %	1.00	0.91	0.82
Calcium, %	0.92	0.90	0.89
Available phosphorus, %	0.45	0.45	0.38
Sodium, %	0.22	0.21	0.20
Chloride, %	0.39	0.34	0.35

*^a^Trace mineral premix added at this rate yields 149.6 mg manganese, 55.0 mg zinc, 26.4 mg iron, 4.4 mg copper, 1.05 mg iodine, 0.25 mg selenium, a minimum of 6.27 mg calcium, and a maximum of 8.69 mg calcium per kilogram of diet. The carrier is calcium carbonate, and the premix contains less than 1% mineral oil*.

*^b^Vitamin premix added at this rate yields 11,023 IU vitamin A, 3,858 IU vitamin D_3_, 46 IU vitamin E, 0.0165 mg B_12_, 5.845 mg riboflavin, 45.93 mg niacin, 20.21 mg d-pantothenic acid, 477.67 mg choline, 1.47 mg menadione, 1.75 mg folic acid, 7.17 mg pyroxidine, 2.94 mg thiamine, 0.55 mg biotin per kilogram diet. The carrier is ground rice hulls*.

*^c^Diamond V Original XPC, Cedar Rapids, IA, USA*.

### Sample Collection

In order to evaluate the effect of dietary XPC on the lymphocyte repertoire in broilers, five randomly selected chickens from each group were euthanized and immune organs (bursa, thymus, spleen, and blood) were harvested at 14, 21, 28, and 42 days of age. Half of each organ was processed for further lymphocyte isolation, while the other half was stored in RNAlater^®^ (Life Technologies, Inc., Carlsbad, CA, USA) for gene expression profiling (1 mg tissue sample to 1 mL stabilization solution ratio). RNAlater^®^ immersed samples were stored at −20°C until further analysis.

### Single Cell Suspension Preparation and Lymphocyte Isolation

Lymphocytes from spleen, bursa, and thymus were prepared according to previous published protocols with minor modifications (11). Briefly, freshly isolated immune organs were passed through 70 μM mesh cell strainers and lymphocytes, including mononuclear cells, were isolated by density gradient sedimentation using Ficoll-Hypaque (Histopaque-1077, Sigma-Aldrich, St. Louis, MO, USA). Isolated single cell suspensions were kept on ice until they were immunofluorescently stained for surface markers.

Similar procedures were applied to PBMC isolation (11). Briefly, 3 mL of peripheral blood was collected from the brachial vein in sodium citrate-containing tubes. PBMC were further isolated by density gradient sedimentation method as previously described. Isolated PBMC were kept on ice until immunofluorescent staining was performed.

### Lymphocyte Subset Analysis by Flow Cytometry

All the primary monoclonal antibodies were purchased from Southern Biotech (Birmingham, AL, USA). Freshly prepared single cell suspensions were treated with 25 mg/mL of mouse whole molecule IgG (Jackson Immunoresearch, West Grove, PA, USA) on ice for 30 min to avoid non-specific Fc receptor binding. Four-color immunostaining with FITC-conjugated-anti-CD3 (0.5 mg/mL), R-PE-conjugated anti-CD4 (0.1 mg/mL), Cy5-conjugated anti-CD8 (0.1 mg/mL), and biotinylated Bu-1 (0.5 mg/mL) were adapted for the current study. Single-color staining was used for compensation of overlapping spectra. After staining with dye-conjugated primary antibodies, all samples were centrifuged at 1,500 × *g* for 5 min at 4°C and washed three times with fluorescence-activated cell sorting (FACS) buffer (10% FBS and 0.1% sodium azide in PBS, pH 7.4). After washing, 0.5 μg/mL of Pac-Blue conjugated streptavidin (Jackson ImmunoResearch) was added and incubated in the dark for 15 min at 4°C, then washed 2 times with FACS buffer. The stained samples were re-suspended with FACS buffer and fixed with 2% (w/v) phosphate-buffered paraformaldehyde. Data were acquired on a BD FACSAria II cell sorter system (BD Biosciences, San Jose, CA, USA) and analyzed with FlowJo version 8.8.4 software (Tree Star, Ashland, OR, USA). Results were analyzed by performing the Student’s *t*-test between control and XPC group using JMP software (SAS Institute, Cary, NC, USA). Statistical significance was determined at *P* < 0.05.

### Gene Expression Analysis by Quantitative PCR Array

Total RNA was extracted from bursa, thymus, and spleen using the Trizol^®^ method according to manufacturer’s instructions. Total RNA samples were then converted into cDNA using the RT2 First Strand Kit (SABiosciences, Frederick, MD, USA). cDNA was then added to the RT2 SYBR Green Master Mix (SABiosciences, Frederick, MD, USA). All mixtures were applied on the Chicken Innate & Adaptive Immune Responses RT2 Profiler PCR Array (SABiosciences, Frederick, MD, USA). All steps were done according to manufacturer’s instructions for the ABI 7900HT Fast Real Time PCR System. The data were analyzed using Excel-based PCR array Data Analysis Templates. Gene expression differences between control and XPC group with a *P*-value < 0.05 and |Fold Change (FC)| > 1.2 were considered as significantly influenced by XPC supplementation.

## Results

### The Effect of *S. cerevisiae* Fermentation Product Supplementation on Cellular Immunity Parameters: T- and B-Cell Repertoire in Immune Organs

**Figure 1:**
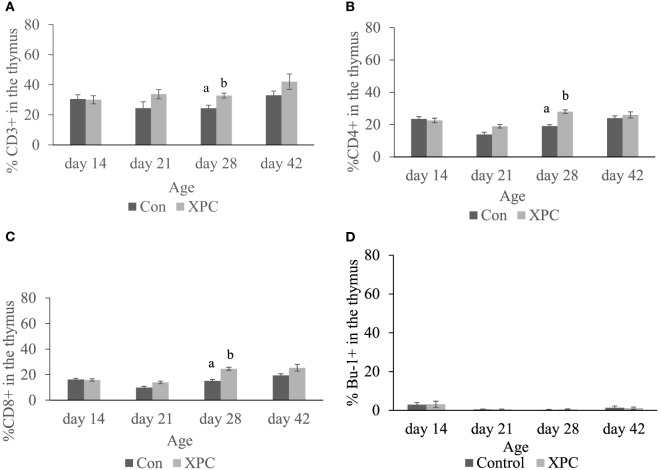
**Lymphocyte subpopulations in thymus at 14, 21, 28, and 42 days of age**. Percentage of **(A)** CD3^+^; **(B)** CD4^+^; **(C)** CD8^+^; **(D)** Bu-1^+^ lymphocytes in control (dark column) and XPC-supplemented (gray column) broilers (1.25 kg/ton; Diamond V Original XPC, Cedar Rapids, IA, USA). *n* = 5 per treatment per day. Data are presented as group means ± SEM. Different letters indicate significant differences (*P* < 0.05).

As shown in Figure 1, XPC supplementation significantly (*P* < 0.05) increased the T-cell repertoire (CD3^+^, CD4^+^, and CD8^+^ T-cells) in the thymus at 28 days of age, 7 days post-NDV vaccine boost. However, at 21 and 42 days of age, only numerically higher percentages of CD3^+^, CD4^+^, and CD8^+^ T-cells were observed in the thymus of XPC supplemented broilers (Figure 1).

**Figure 2:**
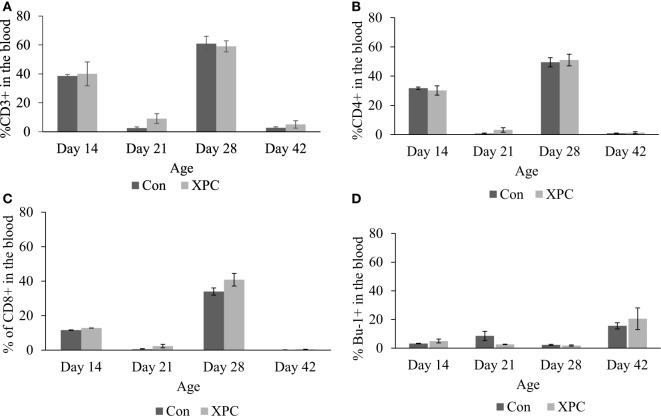
**Lymphocyte subpopulations in blood at 14, 21, 28, and 42 days of age**. Percentage of **(A)** CD3^+^; **(B)** CD4^+^; **(C)** CD8^+^; **(D)** Bu-1^+^ lymphocytes in control (dark column) and XPC-supplemented (gray column) broilers (1.25 kg/ton; Diamond V Original XPC, Cedar Rapids, IA, USA). *n* = 5 per treatment per day. Data are presented as group means ± SEM. Different letters indicate significant differences (*P* < 0.05).

The effect of dietary XPC on lymphocyte subpopulations in peripheral blood was not significant (Figure 2). However, the proportional change in T-cell marker (CD3 and CD4) staining patterns mirrors NDV-specific antibody titers (10) that were measured in the same birds. The proportion of positive staining T-cells dramatically increased (*P* < 0.05) at 14 and 28 days of age and decreased at 21 and 42 days of age.

**Figure 3:**
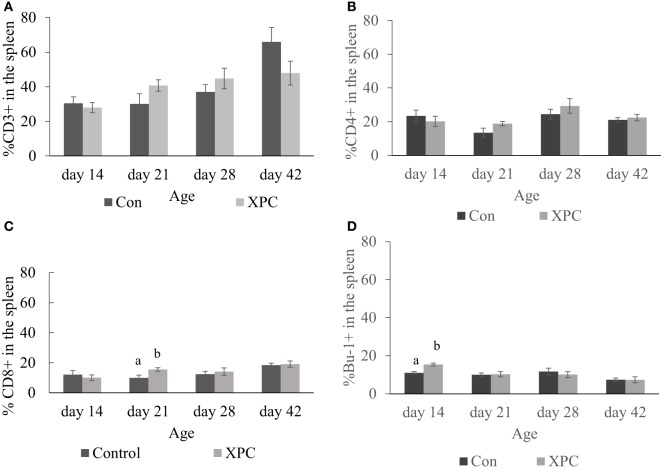
**Lymphocyte subpopulations in spleen at 14, 21, 28, and 42 days of age**. Percentage of **(A)** CD3^+^; **(B)** CD4^+^; **(C)** CD8^+^; **(D)** Bu-1^+^ lymphocytes in control (dark column) and XPC-supplemented (gray column) broilers (1.25 kg/ton; Diamond V Original XPC, Cedar Rapids, IA, USA). *n* = 5 per treatment per day. Data are presented as group means ± SEM. Different letters indicate significant differences (*P* < 0.05).

In the spleen, dietary XPC did not significantly change T-cell subpopulations. As shown in Figure 3, birds supplemented with XPC had a significantly (*P* < 0.05) higher percentage of CD8^+^ T-cells positive staining at 21 days of age. The number of Bu-1^+^ B-cells was significantly (*P* < 0.05) higher in the spleen of birds supplemented with XPC at 14 days of age.

**Figure 4:**
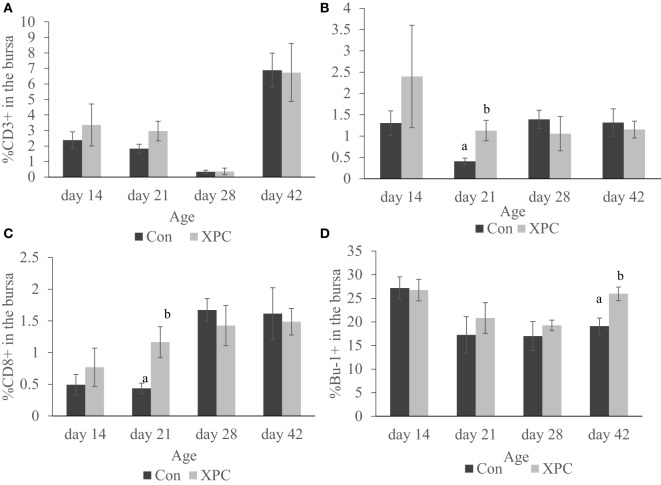
**Lymphocyte subpopulations in bursa at 14, 21, 28, and 42 days of age**. Percentage of **(A)** CD3^+^; **(B)** CD4^+^; **(C)** CD8^+^; **(D)** Bu-1^+^ lymphocytes in control (dark column) and XPC-supplemented (gray column) broilers (1.25 kg/ton; Diamond V Original XPC, Cedar Rapids, IA, USA). *n* = 5 per treatment per day. Data are presented as group means ± SEM. Different letters indicate significant differences (*P* < 0.05).

As shown in Figure 4, the supplemented broilers had a significantly (*P* < 0.05) higher percentage of CD4^+^ and CD8^+^ T-cells at 21 days of age in the bursa. The number of Bu-1^+^ B-cells in the bursa was also significantly (*P* < 0.05) higher at 42 days of age.

### Gene Expression Analysis Using Quantitative PCR Array

**Figure 5:**
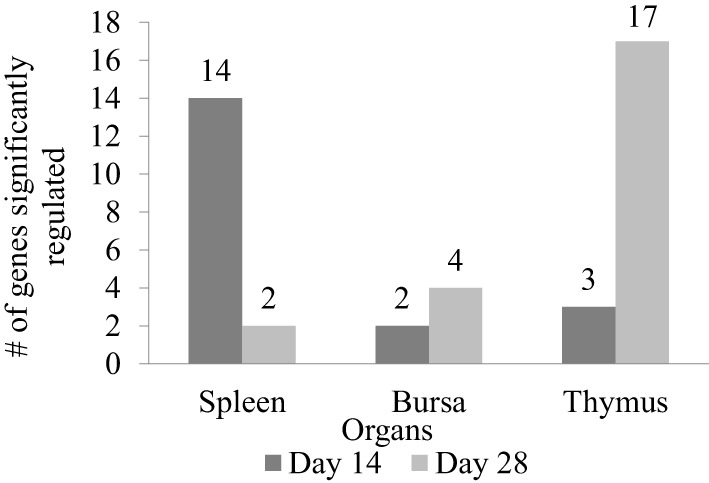
**Number of genes significantly regulated between XPC feed additive group and control in immune organs on the host response to a live Newcastle disease virus vaccine strain *in vivo***. Immune-associated gene expression pattern in spleen, bursa, and thymus between XPC and control groups measure by qPCR array on days 14 and 28 of age. Fold regulation more than 1.2 and *P* < 0.05 was considered as significantly regulated.

In order to measure the effects of XPC feed additive on the host response to a live NDV vaccine strain *in vivo*, a qPCR array covering 84 immune-associated genes was performed. Tables 2–4 summarize the significantly modulated (*P*-value < 0.05 and |FC| > 1.2) genes in the thymus, spleen, and bursa of Fabricius of the controls and the XPC-supplemented birds. As shown in Figure 5, immune-associated gene expression patterns were different between central and peripheral immune organs. At 14 days of age, the spleen was the major immune organ that was significantly modulated by XPC. There were 14 genes significantly (*P* < 0.05) modulated in the spleen. On the other hand, only two and three genes were significantly (*P* < 0.05) modulated in the bursa and thymus, respectively (Figure 5).

**Table 2:** **Genes significantly up- or downregulated in the thymus with supplementation of XPC**.^a,b^

Thymus	Day 14		Day 28	
Gene	Fold regulation (XPC vs. control)	*P*-value	Fold regulation (XPC vs. control)	*P*-value
CD14	–	–	−1.5199	0.0274
CD80	–	–	1.2924	0.0013
CD86	–	–	1.631	0.0466
CXCL12	–	–	−1.5959	0.0311
FAS	–	–	1.95	0.0033
IFIH1	–	–	1.4894	0.0251
IFNAR1	–	–	1.3719	0.0431
IL10	–	–	1.436	0.0349
IL15	–	–	1.5614	0.0002
IL1R1	–	–	1.8698	0.0205
IL2	–	–	1.7623	0.0146
IL8L1	–	–	2.2771	0.0051
IRF1	–	–	1.4805	0.0199
ITGB2	1.5158	0.0098	–	–
Mitogen-activated protein kinase 8	−1.397	0.0268	–	–
MX1	–	–	2.9625	0.0453
Myeloid differentiation primary response gene 88	−2.376	0.0076	–	–
PTGS2	–	–	1.6087	0.0455
STAT1	–	–	1.6156	0.0038
TLR3	–	–	1.6099	0.0051

*^a^Diamond V Original XPC, Cedar Rapids, IA, USA. Supplemented in the diet at 0 (control) or 1.25 kg/ton of feed*.

*^b^Fold regulation is the fold change (upregulation as a positive value or downregulation as a negative value) of genes in birds supplemented with XPC compared to control. A blank value indicates no differences between the two groups of birds*.

**Table 3:** **Genes significantly upregulated in the spleen with supplementation of XPC**.^a^^,^^b^

Spleen	Day 14		Day 28	
Gene	Fold regulation (treated vs. control)	*P*-value	Fold regulation (treated vs. control)	*P*-value
C3	7.3613	0.0179	–	–
CASP8	1.3845	0.0328	–	–
CRP	–	–	2.7613	0.0086
FAS	3.119	0.0231	–	–
FASLG	–	–	1.5077	0.0203
IFIH1	2.2836	0.0489	–	–
IFNAR1	1.5519	0.0257	–	–
IFNG	3.0904	0.0270	–	–
IL17C	1.6033	0.0473	–	–
LY96	2.3354	0.0280	–	–
Mitogen-activated protein kinase 8	2.4213	0.0185	–	–
MBL2	5.1335	0.0224	–	–
NFKB1	1.4967	0.0147	–	–
NOD1	1.3814	0.0014	–	–
Signal transducer and activator of transcription 4	2.3667	0.0026	–	–
TLR2-2	1.9828	0.0028	–	–

*^a^Diamond V Original XPC, Cedar Rapids, IA, USA. Supplemented in the diet at 0 (Control) or 1.25 kg/ton of feed*.

*^b^Fold regulation is the fold change (upregulation as a positive value or downregulation as a negative value) of genes in birds supplemented with XPC compared to control. A blank value indicates no differences between the two groups of birds*.

**Table 4:** **Genes significantly up- or downregulated in the bursa with supplementation of XPC**.^a^^,^^b^

Bursa	Day 14		Day 28	
Gene	Fold regulation (treated vs. control)	*P*-value	Fold regulation (treated vs. control)	*P*-value
CASP1	−1.2477	0.0397	–	–
CD28	–	–	−1.202	0.0341
CRP	–	–	2.05	0.0293
CSF2	1.5786	0.0429	–	–
JAK2	–	–	1.4361	0.0103
TLR21	–	–	−2.7123	0.0186

*^a^Diamond V Original XPC, Cedar Rapids, IA, USA. Supplemented in the diet at 0 (control) or 1.25 kg/ton of feed*.

*^b^Fold regulation is the fold change (upregulation as a positive value or downregulation as a negative value) of genes in birds supplemented with XPC compared to control. A blank value indicates no differences between the two groups of birds*.

Interestingly, at 28 days of age (1 week post-boost of the vaccination), the pattern of gene expression changed entirely. Thymus became the major immune organ that was significantly modulated by XPC supplemented. Seventeen genes were significantly (*P* < 0.05) modulated in the thymus. However, only 2 and 4 genes were significantly (*P* < 0.05) regulated in the spleen and bursa, respectively (Figure 5).

### Genes Differentially Expressed in the Immune Organs of XPC-Supplemented Broilers at 14 Days of Age

At 14 days of age, 2 weeks after the primary NDV vaccination, only three and two genes were significantly (*P* < 0.05) modulated in the thymus and bursa of Fabricius of the XPC group when compared with control groups, respectively (Tables 2 and 4). The most affected upregulated gene in the thymus at 14 days of age was *Integrin* β*2 (ITGB2*) (Table 2). Conversely, two genes, mitogen-activated protein kinase (MAP kinase) and myeloid differentiation primary response gene (MyD88), were downregulated in the thymus at 14 days of age (Table 2).

As shown in Table 3, our results revealed 14 genes in the spleen that were significantly (*P* < 0.05) affected in the treated group on 14 days of age, which involved three different functional categories: innate immunity, antiviral, and pro-inflammatory. The most affected gene affected by XPC supplementation, complement component 3 (C3), was sevenfold upregulated over the control group. C3 also plays a role in B-cell activation and is thought to facilitate adaptive immunity (12, 13). Several germline-encoded innate immune sensors are among the significantly modified genes of the XPC group, including toll-like receptors (*TLRs*), interferon induced with helicase C domain 1 (*IFIH1*), and nucleotide-binding oligomerization domain containing 1 (*NOD1*) (Table 3). IFN-stimulated genes and transcription factors, such as interferon (alpha, beta, and omega) receptor 1 (*IFNAR1*), *IFN-*γ, STAT4, and *NF*κ*B*, were also upregulated in the spleen of XPC-supplemented broilers. Two genes involved in apoptosis, Caspase 8 and apoptosis-related cysteine peptidase (*CASP8*), and Fas TNF receptor superfamily, member 6 (*FAS*) were also upregulated in the spleen of the XPC group at 14 days of age. Gene of cytokine IL-17, produced by T-helper (T_h_) 17 cells, was also significantly upregulated in the spleen of XPC-supplemented broilers at 14 days of age (Table 3).

### Genes Differentially Expressed in Immune Organs of XPC Supplemented Broilers at 28 Days of Age

At day 28 of age (1 week post-boost of the vaccination), 17 (15 up- and 2 downregulated) and 4 (2 up- and 2 downregulated) genes were differentially expressed (*P* < 0.05) in the thymus and bursa of Fabricius, respectively, in XPC-supplemented broilers when compared with control (Tables 2 and 4). As shown in Table 2, several IFNs, IFN-stimulated genes, and transcription factors [such as *IFIH1, IFNAR1*, Myxovirus resistance 1 (*Mx1*), Interferon regulatory factor (*IRF*) 1, *IRF7*, and *STAT1*] were significantly (*P* < 0.05) upregulated in the thymus of the XPC group at 28 days of ages. Several cytokines that contribute to lymphocyte proliferation and differentiation (including *IL-1R1, IL-2, IL-10*, and *IL-15*) were also significantly (*P* < 0.05) upregulated in the thymus of broilers supplemented with dietary XPC at 28 days of age (Table 2). Two genes [Chemokine (C–X–C motif) ligand 12 (stromal cell-derived factor 1) *CXCL12* and *CD14*] were significantly (*P* < 0.05) downregulated in the thymus of the XPC group at 28 days of age when compared with the control group. CXCL12 is a chemokine produced by stromal cells and plays a pivotal role in the migration and localization of developing T-cells within different zones of the thymus (Table 2).

## Discussion

In the current study, the live-attenuated NDV vaccination model on broilers was adopted. The effect of *S. cerevisiae* fermentation product (Original XPC™, Diamond V) as a natural immunomodulator on central and peripheral immune organs was evaluated at the cellular and molecular levels.

The differentially expressed genes (*P* < 0.05) at 14 and 28 days of age were grouped into different functional groups. Interestingly, several distinct modes of action were observed. Several germline-encoded innate immune sensors were affected by XPC supplementation. Pathogens that invade a host are initially recognized by the innate immune system through germline-encoded pattern recognition receptor (PRRs). TLRs, NOD-like receptors, and RIG-I-like proteins (RLRs) are major classes of PRRs with key roles in immune homeostasis and defense against infections (14–16). Stimulation of PRRs by pathogen-associated molecular patterns plays a crucial role in shaping the profile of the subsequent adaptive immune responses. In the current study, our results revealed that 2 weeks post primary vaccination with the NDV B1 strain, a stronger expression of TLR-2, NOD-1, and IFIH-1 was measured in the spleen of XPC-supplemented broilers.

Yitbarek et al. (17) reported broilers fed 0.02% yeast-derived macromolecule supplements showed TLR2 and TLR4 gene upregulation in the spleen at 42 days of age when compared with control. Our current findings reveal that when challenged with live-attenuated NDV vaccine, broilers fed XPC significantly upregulated TLR2, but not TLR4. This inconsistency could result from the NDV vaccine challenge. TLR3, NOD-1, and IFIH-1 are common host sentinel proteins for NDV (18). In this study, NOD-1 and IFIH-1 were significantly upregulated in the spleen 2 weeks post primary vaccination in the XPC group. TLR3 was also upregulated despite not reaching statistical significance (*P* = 0.07).

In the chicken, TLR2 is widely distributed in the heart, liver, gizzard, muscle, spleen, cecal tonsils, bursa of Fabricius, and liver (19). TLR2 has also been detected by RT-PCR in heterophils, monocytes, macrophages, B-cells, and T-cells (20, 21). The ligation of TLR2 with various ligands can trigger different immunological events (22). In mammals, zymosan, a *S. cerevisiae* cell wall derivate, is recognized by TLR2 and induces peripheral tolerogenic responses *via* induction of regulatory antigen-presenting cells [APCs; (22, 23)]. Thus, the upregulation of TLR2 by XPC supplementation in broilers may contribute to maintaining the balance of the immune system and preventing innate immunity over-reaction in the host.

In the current study, NOD1 and MDA-5 in the spleen were able to recognize NDV through CARD-dependent recruitment of RIP2, driving activation of MAP kinase and NF-κB. These observations are in line with results from previous studies (18). At 14 days of age, both type I (IFN-α, *P* = 0.07 and IFN-β, *P* = 0.07) and type II (IFN-γ) IFN responses were detected by qPCR array in the spleen of XPC-supplemented broilers. These cytokines may participate in priming natural killer (NK) cells, inducing maturation of Th1 cells, and viral clearance (24, 25). IFN-γ is also involved in leukocyte attraction, regulation of B-cell immunoglobulin production, and class-switching (26). These results are consistent with the recent report by Yitbarek et al. (17) where the authors showed yeast-derived macromolecules significantly enhanced IFN-γ in spleen compared with non-supplemented control birds. However, the up-stream signal interferon regulatory factor-1 and 7 were not significantly altered in the spleen of XPC-supplemented broilers. The downstream effectors of IFN action include Mx1 and MyD88, and those genes were not consistently modulated (*P* > 0.05). We also noticed that IL-17, a Th17 cell-produced cytokine, was significantly upregulated in the spleen of XPC-supplemented broilers. Th17 cells are important in clearing pathogens during host defense reactions (27). IL-17 is also a factor that contributes to the formation of germinal centers of lymphoid follicles (28), which is consistent with our flow cytometry data that XPC-supplemented broilers have higher percentages of Bu-1^+^ B-cells in the spleen at 14 days of age. These results suggest that XPC-supplemented broilers have higher baseline expression of IFNs and can keep the innate immune system in stand-by mode. As a consequence, supplemented broilers are capable of responding to stimuli more rapidly than their non-treated counterparts.

In our previous study (10), XPC supplementation was able to establish robust NDV-specific humoral immunity against an NDV vaccine challenge at 28 days of age, which is 1 week earlier than control birds. Since the spleen is the most important peripheral lymphoid organ in chickens, we expected to observe more conspicuous effects immune-associated genes at this time point. Interestingly, our gene expression data revealed that at the same time point, more genes were affected in the thymus and not in the spleen. It is possible that the timing of that sampling point was not optimal. Rue et al. (29) reported that a virulent NDV-CA02 challenge elicited a strong innate immune response within 72 h in the spleen of specific pathogen-free chicken (29). Despite broilers in the current study receiving a live-attenuated NDV LaSota strain booster, after 7 days the acute effects of the viral challenge may be over. The gene expression profile in the thymus of XPC-supplemented broilers at 28 days of age, in spite of the fact that no significant IFN regulation was observed, showed enhanced expression of the type I IFN receptor. The activation of type I IFN receptor results in the induction of IRF 1 and 7 (*P* = 0.056) required for regulation of the downstream interferon stimulatory gene. In this study, the expression of *Mx1* gene, important for resistance against viral infection, and *STAT1* were significantly induced. IFNs are a family of cytokines that play a central role in innate immunity to viruses. In the thymus, IFNs are constitutively expressed in the medulla and have a direct impact on the development of T-cells in the thymus (16). The significantly upregulated IFN-associated genes may contribute to innate immune responses and also may shape the repertoire of T-cells in the thymus, which is consistent with our flow cytometric data.

In the present study, several cytokines were significantly upregulated in the thymus of XPC-supplemented broilers at 28 day of age. In mammals, IL-1R expression in the thymus is restricted on immature T-cells. However, the function of IL-1R in avian species is still unknown (30). IL-2 is important for the production, function, and homeostasis of CD4^+^CD25^+^ T_reg_ cells. It is also a potent T-cell growth factor during the initiation of immune responses (31, 32). IL-10 is a pleiotropic cytokine. The expression of IL-10 plays a critical role in limiting immune response to pathogens, including downregulation of T_h_1 cytokines, MHC-molecules, and co-stimulatory molecules on macrophages (33). Taken together, the significant upregulation of IL-2 and IL-10 in the thymus of supplemented broilers at 28 days of age indicates XPC promotes the developmental production of suppressive T_reg_ cells and suppresses the innate immune response, thus contributing to the proportional, i.e., transient nature, of the innate immune responses induced by NDV vaccine challenge. IL-15, a cytokine essential for NK cell development, functional maturation and activation was significantly upregulated in supplemented broilers. This result is in concert with previous studies showing XPC treatment induced activation markers CD69 and CD25 on CD3^+^–CD56^+^ NK cells in mammals (34). CXCL12 is produced by thymus stromal cells in the cortex and play a pivotal role guiding the traffic of T-cell precursors during early development (35, 36). The downregulation of CXCL12 in the XPC group indicates XPC supplement accelerated the development of T-cells compared with the control group. This result is also consistent with our flow cytometric data showing significantly more CD4^+^ and CD8^+^ T-cells in the thymus of the XPC group at 28 days of age.

CMI is a crucial factor for development of protection and viral clearance as well as vaccine-induced protective immunity in mammals and chickens (37). The subpopulations of T-cells, including cytokine-secreting CD4^+^ T-helper cells and CD8^+^ cytotoxic T-cells (CTL), constitute the main components of the CMI responses.

Our data indicate that dietary XPC increased the repertoire of both effector T-cells [T-helper (CD4^+^) and Cytotoxic (CD8^+^)] in the thymus 1 week after NDV vaccine challenge. The thymus has been shown to be a sensitive immune organ following exposure to immune toxins and endogenous corticosteroids, especially the cortex T-cells. An increase in lymphocyte numbers in the thymus is usually a response to antigenic stimulation, and the cell population is mixed in contrast to the more homogeneous neoplastic population. The thymus is a primary lymphoid organ, able to generate mature T-cells that eventually colonize T-cell-dependent areas in secondary lymphoid organs, and is essential for peripheral T-cell renewal. Higher percentages of effector T-cells in the thymus signify a more diverse T-cell repertoire to aid in the host’s fight against pathogens. Therefore, XPC-supplemented broilers are more capable of establishing protective immunity thanks to antigen-specific T-cell expansion, further facilitating subsequent proliferation and differentiation of CD4^+^ T_h_ cells and CD8^+^ CTL. In the current study, the output of naïve T-cells may have contributed to the NDV vaccine-induced humoral immune response. These results are consistent with our previous report that NDV-specific antibody titers peak 1 week post-boost [28 days of age; (10)]. We expect matured naïve T-cells in the thymus to migrate to the periphery and to contribute to the shaping of the lymphocyte distribution in circulation and spleen. However, no differences between supplemented broiler and non-supplemented control were observed in the percentage of effector T-cells and B-cells in the circulation across all the experiment sampling time points. As previously mentioned, sampling 7 days post-boost may be too late for measuring the dynamic change of lymphocyte subpopulations in the circulation and the spleen.

Also, our data show that inclusion of XPC into the feed significantly (*P* < 0.05) increased the percentage of Bu-1^+^ B-cells in the spleen at 14 days of age. Bu-1 marker is expressed on early and mature B cells, except plasma cells (38). Plasma cells are effector B-cells that can secrete antibodies. Our current study suggests that the higher percentage of Bu-1^+^ B-cells in the spleen indicates XPC promotes the growth and maturation of B-cells in the spleen. These results are consistent with our previous finding that supplemented broilers have more white pulp in the spleen at 14 days of age (10).

Furthermore, supplementation with XPC significantly increased the percentage of CD4^+^ and CD8^+^ in the bursa at 21 days of age. Also, significantly higher percentages of Bu-1^+^ B-cells were measured in the supplemented group at 42 days of age. The bursa of Fabricius is a unique primary lymphoid organ where B-cell lymphopoiesis takes place (39). Higher B-cell proliferation rates were seen in the supplemented group, indicating a more diverse B-cell repertoire. This might also contribute to enhancement of the humoral immune response.

Only very limited resident T-cells reside in the uninfected bursa. How the inclusion of XPC affects the T-cell population in the bursa at 21 days of age and how it contributes to the immune response against NDV vaccine requires further investigation.

Our current study suggests inclusion of XPC in the feed modulates more than one aspect of the coordinated immune response to NDV vaccine challenge through activation of genes involved in the innate immune response and moderation of potentially excessive immune responses. More importantly, XPC triggers the innate immune system mildly, thereby priming the defense mechanisms, thus shortening the time for establishing the adaptive immune response.

## Ethics Statement

This study was performed at Texas A&M University Poultry Research, Teaching and Extension Center (TAMUPRC) and was conducted under TAMU approved animal use protocol IACUC 2014-0017.

## Author Contributions

WC was responsible for a majority of the lab work, data analysis and interpretation. JP was the graduate student in the project and provided bird care and lab work as well as some data analysis. JC was CO-PI and provided project oversight, experimental design, data analysis, and interpretation. DM provided technical details of the product, assisted with experimental design. LB was CO-PI and provided experimental design, data analysis, and interpretation.

## Conflict of Interest Statement

The authors declare that the research was conducted in the absence of any commercial or financial relationships that could be construed as a potential conflict of interest.
